# Management of Patients with Suspected Hypersensitivity to Chemotherapy Drugs: A Practical Approach in a Tertiary Care Setting

**DOI:** 10.3390/mps9020056

**Published:** 2026-04-02

**Authors:** Maria Bernadette Cilona, Serena Nannipieri, Arianna Ferlito, Giulia Orsi, Monica Ronzoni, Giorgia Mangili, Emanuela Rabaiotti, Fabio Ciceri, Michele Reni, Mona-Rita Yacoub

**Affiliations:** 1 Multidisciplinary Advanced Center of Asthma, Food and Drug Allergy, IRCCS San Raffaele Hospital, 20132 Milan, Italy; cilona.maria@hsr.it (M.B.C.); nannipieri.serena@hsr.it (S.N.); ferlito.arianna@hsr.it (A.F.); 2 Oncology Unit, Pancreas Translational and Clinical Research Center, IRCCS San Raffaele Scientific Institute, 20132 Milan, Italy; orsi.giulia@hsr.it (G.O.); reni.michele@hsr.it (M.R.); 3 Unit of Oncology, IRCCS San Raffaele Scientific Institute, 20132 Milan, Italy; ronzoni.monica@hsr.it; 4 Unit of Gynecology and Obstetrics, IRCCS San Raffaele Scientific Institute, 20132 Milan, Italy; mangili.giorgia@hsr.it (G.M.); rabaiotti.emanuela@hsr.it (E.R.); 5 Hematology and Bone Marrow Transplant Unit, IRCCS San Raffaele Scientific Institute, 20132 Milan, Italy; ciceri.fabio@hsr.it

**Keywords:** desensitization, chemotherapy-induced hypersensitivity, anaphylaxis, skin testing

## Abstract

Background: Hypersensitivity reactions to antineoplastic agents are an increasing clinical challenge due to their rising incidence and potential severity. Early allergological assessment and tailored drug re-exposure strategies may allow continuation of essential therapies, although real-world data remain limited. Methods: We conducted a monocentric retrospective observational study including adult oncologic patients with suspected chemotherapy-induced hypersensitivity reactions. Clinical features, allergological work-up, and management strategies were analyzed. The primary outcome was the success rate of drug reintroduction using desensitization or enhanced premedication. Secondary outcomes included skin test positivity rates and the association between cumulative chemotherapy exposure and anaphylaxis. Results: Forty-two patients were included (95% female; median age 57.5 years). Re-exposure was required in 18 patients, and was successful in all patients undergoing desensitization and in 92% of those managed with enhanced premedication. Skin testing was positive in 71% of suspected platinum reactions, 30% of taxanes, and 40% of biologic agent reactions. Anaphylaxis occurred in 26.2% of patients, and a trend toward an association with cumulative chemotherapy exposure was observed; each additional cycle was associated with a 28% increase in the odds of anaphylaxis (adjusted OR 1.28; 95% CI, 1.00–1.63). Conclusions: Desensitization and enhanced premedication allow safe reintroduction of antineoplastic agents. Cumulative chemotherapy exposure is associated with an increased risk of anaphylaxis.

## 1. Introduction

Chemotherapy-related hypersensitivity reactions (HRs) represent a growing concern in the field of oncology due to their rising incidence, potential severity, and impact on cancer treatment and patient outcomes [1]. In 2022, about 20 million new cancer cases were diagnosed worldwide and approximately 9.7 million deaths were attributed to cancer, according to data from the International Agency for Research on Cancer (IARC) [2]. Around one-third of cancer patients undergo chemotherapy as part of their first-line treatment, and approximately 5% of them experience hypersensitivity reactions to antineoplastic agents. Platinum salts, taxanes, and monoclonal antibodies are among the most implicated agents and account for most severe reactions, particularly in patients with gynaecologic, breast, colorectal, and pancreatic malignancies [3]. Chemotherapy-related HRs often occur unpredictably, ranging from mild cutaneous symptoms to life-threatening anaphylaxis, and may arise either at first administration or after multiple treatment cycles [4].

These reactions represent a significant therapeutic challenge, as the culprit drugs frequently represent the first-line or most effective treatments of chemotherapy regimens. The assessment and management of chemotherapy-related HRs require a multidisciplinary approach between allergists, oncologists, and infusion centre nurses [5]. Accurate diagnosis requires a tailored allergological work-up, combining clinical history, skin testing, in vitro assays, and, in selected cases, drug tests. When the implicated chemotherapeutic agent cannot be replaced, drug desensitization allows modulating mast-cell and basophil reactivity through controlled, stepwise drug exposure. Indeed, desensitization induces a reversible tolerant state that allows safe drug reintroduction under strict monitoring [6]. Additionally, premedication strategies reduce the risk of adverse reactions while reintroducing culprit or related antineoplastic agents in patients with assessed low risk of previous hypersensitivity reactions [7]. Despite increasing evidence supporting that rapid desensitization and premedication strategies ensure the most appropriate treatment strategy, their implementation remains inconsistent outside tertiary care centres. In smaller hospitals, where collaboration between oncologists and allergists is limited and access to specialized diagnostic testing and desensitization strategies is lacking, clinicians frequently switch to alternative regimens with potential impacts on outcomes of disease progression [8].

In this context, we conducted a retrospective study aimed at identifying success rate of drug reintroduction with desensitization or enhanced premedication, describing the clinical features, diagnostic work-up, and management outcomes of patients with suspected chemotherapy-induced HRs referred from oncologists to our multidisciplinary Allergology unit.

## 2. Materials and Methods

### 2.1. Study Design and Setting

We conducted a monocentric, retrospective observational study at the Allergy Unit of San Raffaele Hospital, Milan, a tertiary referral centre with specialized expertise in the diagnosis and management of chemotherapy-induced HRs. The study period ranged from November 2023 to November 2024. The study protocol was approved by the Institutional Review Board of San Raffaele Hospital. Informed consent was waived due to the retrospective design. All patient data were anonymized to ensure confidentiality.

### 2.2. Participants

Eligible participants were adult oncologic patients (aged ≥ 18 years) referred for suspected drug hypersensitivity reactions temporally associated with the administration of antineoplastic agents. Patients were included if they fulfilled all the following inclusion criteria: (1) a documented hypersensitivity reaction occurring during or within 1 h (immediate) or beyond 1 h (non-immediate) of infusion, and (2) availability of a full allergological assessment, including skin testing, in vitro assays, or re-exposure via desensitization protocols. Patients were excluded if their clinical records were incomplete, if the reaction was clearly attributable to non-chemotherapeutic agents, or if allergological testing was not performed. Only patients with complete clinical documentation and a full allergological assessment were included in the analysis to ensure the reliability of the collected data. All eligible consecutive patients referred to the Allergy Unit during the study period were considered for inclusion in order to minimize potential selection bias.

### 2.3. Definitions and Classification of Reactions

Hypersensitivity reactions were classified as immediate (≤1 h from infusion) or delayed (>1 h) according to European Academy of Allergy and Clinical Immunology/World Allergy Organization (EAACI/WAO) criteria [9]. Reaction severity was graded using Ring and Messmer classification and categorized as mild, moderate, or severe (anaphylaxis) [10]. Clinical manifestations were grouped into cutaneous, respiratory, cardiovascular, gastrointestinal, and multiorgan involvement. Anaphylaxis was defined according to the most recent WAO definition as a serious systemic hypersensitivity reaction that is usually rapid in onset and may cause death with compromised in airway, breathing and/or circulation, and may occur without typical skin features or circulatory shock [11].

### 2.4. Allergological Assessment

All patients underwent a structured allergological evaluation that included a detailed clinical history, assessment of latency, drug culprit suspicion, and previous antineoplastic drug tolerance. Skin prick tests (SPT) and intradermal tests (IDT) were performed according to EAACI/European Network on Drug Allergy (ENDA) standardized concentrations for platinum compounds, taxanes, and biologic agents. A positive result was defined as a wheal diameter ≥ 3 mm compared to negative control [12]. In vitro testing, including serum tryptase, was performed when appropriate. Drug provocation tests were not performed because re-exposure strategies were based on allergological evaluation and multidisciplinary risk assessment. In patients with negative skin test results, the diagnosis of chemotherapy-related HRs was based on clinical assessment, including the temporal relationship between drug administration and symptom onset, the characteristics of the reaction, and the exclusion of alternative causes. In these cases, treatment decisions were made after multidisciplinary discussion, and re-exposure strategies generally involved enhanced premedication protocols when continuation of therapy was clinically required.

### 2.5. Drug Re-Exposure and Desensitization Protocols

In patients for whom the culprit drug was clinically indispensable for oncologic reasons, re-exposure was considered based on skin test results, severity of index reaction, and multidisciplinary risk assessment. The choice between desensitization and enhanced premedication was determined according to the results of the allergological work-up and the severity of the index reaction. Desensitization protocols were applied for IgE-mediated reactions and mild to moderate anaphylactic reactions with positive skin tests to the culprit and alternative drug. Protocols consisted of multistep, incremental dosing over 12–16 steps under continuous monitoring. Conversely, enhanced premedication regimens were adopted in patients with negative skin tests and clinical features suggestive of non-IgE-mediated reactions. Enhanced premedication regimens, including administration of a first-generation H1-antihistamine (chlorpheniramine), intravenous hydrocortisone at a dose of 500 mg, and, in patients with suspected underlying asthma or bronchial hyperreactivity, pre-infusion administration of the leukotriene receptor antagonist, were used in patients with negative skin tests and non-IgE-mediated reactions [13]. During chemotherapy administration, patients routinely received premedication according to standard institutional oncology protocols for the specific antineoplastic regimen. These commonly included corticosteroids, antihistamines, and antiemetic agents administered to reduce the risk of infusion-related reactions and treatment-related adverse effects. These supportive treatments were prescribed according to the oncology protocol and were not specifically modified prior to the occurrence of hypersensitivity reactions.

### 2.6. Data Collection

Demographic, oncologic, and clinical data were extracted from electronic medical records. The number of prior treatment cycles, latency from index reaction to allergological evaluation, skin test outcomes, in vitro assay results, and outcomes of re-exposure (tolerated, breakthrough reactions, treatment discontinuation) were recorded.

### 2.7. Outcomes

Primary outcome was the success rate of drug reintroduction with desensitization or enhanced premedication strategies. Secondary outcomes were the prevalence of positive skin tests to platinum salts, taxanes, and biologics, the rate of adjusted infusion protocols, and the association between the number of prior chemotherapy cycles and the risk of developing anaphylaxis.

### 2.8. Statistical Analysis

Continuous variables were examined for normality using the Shapiro–Wilk test and reported as mean ± standard deviation (SD) or median and interquartile range (IQR), as appropriate [14]. Categorical variables are presented as absolute numbers and percentages. Comparisons between patients with and without anaphylaxis were conducted using the Mann–Whitney U test for non-normally distributed continuous variables and the chi-square or Fisher’s exact test for categorical variables, as applicable [15]. For the exploratory analysis, univariate logistic regression was performed to assess the unadjusted association between number of chemotherapy cycles, age, history of previous drug allergy, and anaphylaxis. Variables with *p* < 0.20 in univariate analysis, as well as those considered clinically relevant, were included in a multivariate logistic regression model to estimate independent predictors. Results are expressed as unadjusted and adjusted odds ratios (ORs) with 95% confidence intervals (CIs). Statistical significance was defined as a two-sided *p*-value < 0.05. Clinical information was systematically extracted from electronic medical records using predefined variables, and only patients with complete allergological evaluation were included in the analysis. In addition, a multivariable logistic regression model was used to adjust for potential confounding factors when assessing the association between clinical variables and the occurrence of anaphylaxis. No imputation was performed for missing data, as all variables included in the analysis were complete.

## 3. Results

### 3.1. Baseline Characteristics

A total of 42 patients with suspected chemotherapy-induced HRs were included. The median age at reaction onset was 57.5 years [50–68]. Of the 42 patients included in the study, 40 (95%) were female and 12 (29%) patients reported prior history of drug allergy. Specifically, four (33%) reported allergy to b-lactams antibiotics, two (17%) to non-steroidal anti-inflammatory drugs (NSAIDS), one (8%) to gemcitabine, three (25%) to taxanes, and one (8%) to doxorubicin. Among patients with a previous drug allergy, three (25%) patients reported previous allergy to antiemetic drugs, and one patient reported previous allergy to allopurinol. Among the 42 patients included, 29 (69%) were treated for gynaecologic cancers, six (14%) for breast cancer, two (5%) for prostate cancer, three (7%) for lung cancer, and two (5%) for colorectal cancer. In 90% of cases, reactions occurred within one hour of administration of the suspected culprit drug and were considered immediate, whereas 10% were classified as delayed, occurring from one hour to four days after suspected culprit drug administration. The suspected culprit agents were predominantly platinum compounds, taxanes, and biologic agents. Among the 42 patients included in the study, 24 hypersensitivity reactions were attributed to platinum compounds, 10 to taxanes, and 5 to biologic agents. These drug classes represent the most frequently implicated antineoplastic agents in chemotherapy-related HRs and were therefore the focus of the allergological evaluation performed in our cohort. Among 42 patients, 26 (62%) developed generalized erythematous rash and ten (24%) pruritus. Four (10%) patients developed extended urticaria and eight (19%) labial and periorbital angioedema. Respiratory involvement was frequent, including dyspnoea in 22 (52%) of patients and acute rhinitis in one case during infusion. Cardiovascular involvement occurred in 9 (21%) patients, characterized by hypotension, tachycardia, and thoracic pain. Gastrointestinal symptoms were reported in 6 (14%) patients with nausea and epigastric pain. Among 42 patients, 11 (26.2%) experienced anaphylaxis. Acute serum tryptase levels were measured in all patients within two hours of symptom onset as part of the diagnostic evaluation of suspected hypersensitivity reactions. The median acute serum tryptase concentration was 5.95 µg/L (4.4–9.5), assessed within two hours from symptom onset. Baseline tryptase levels were not available. Baseline characteristics are reported in Table 1. The culprit drugs and management strategies are summarized in Table 2.

### 3.2. Outcomes

Re-exposure to the culprit or a related chemotherapeutic agent was required in 18 patients. Six patients (33%) underwent a desensitization protocol and twelve (67%) enhanced premedication strategies. In the remaining 24 patients, re-administration of the suspected culprit drug was not pursued because continuation of the same agent was not considered clinically necessary for oncologic treatment, or because effective alternative therapeutic regimens were available. In these cases, allergological evaluation was primarily performed to characterize the hypersensitivity reaction and to guide future treatment decisions if re-exposure became necessary. Among patients undergoing culprit drug desensitization, five experienced mild-grade anaphylaxis. One patient experiencing moderate-grade anaphylaxis underwent alternative related-chemotherapy agent desensitization. Drug reintroduction was successful in all patients undergoing desensitization protocols and 92% undergoing enhanced premedication strategies.

Latency between the hypersensitivity event and allergological assessment ranged from two weeks to six months. Skin testing was performed in 39 cases: of 24 suspected cases of hypersensitivity reactions to platinum compounds, 17 (71%) patients tested positive to skin tests. Among ten patients with suspected allergy to taxanes, three (30%) tested positive to skin tests, while among five patients with suspected allergy to biologic agents 2 (40%) tested positive to skin tests.

Among 42 patients, 11 (26.2%) experienced anaphylaxis. The median number of chemotherapy cycles prior to the hypersensitivity reaction was 2 [1–4.8] in patients without anaphylaxis and 6 [2–9.5] in those with anaphylaxis. Patients who developed anaphylaxis underwent a significantly higher number of previous chemotherapy administrations compared with those without anaphylaxis (*p* = 0.04). In the univariate logistic regression model, only the number of prior chemotherapy cycles was significantly associated with the occurrence of anaphylaxis (*p* = 0.028).

In the multivariate logistic regression model, the number of chemotherapy cycles remained independently associated with the risk of anaphylaxis (adjusted OR, 1.28; 95% CI, 1.00–1.63; *p* = 0.05). Outcomes are reported in Table 3. Regression analysis is reported in Table 4.

## 4. Discussion

In our retrospective study conducted in a multidisciplinary tertiary care setting, we describe the diagnostic evaluation and management of patients with suspected chemotherapy-related HRs. We found that successful drug reintroduction occurred in all patients undergoing desensitization protocols, and 92% undergoing enhanced premedication strategies. In addition, skin testing yielded a higher positivity rate for platinum compounds than taxanes and biologic agents. Our findings highlight that, after allergological assessment, most patients were able to safely continue treatment with the culprit or a related antineoplastic agent using tailored strategies such as desensitization or enhanced premedication. In addition, a trend toward an association between cumulative chemotherapy exposure and the occurrence of anaphylaxis was observed. In both univariate and multivariable analyses, an increasing number of chemotherapy cycles was associated with approximately 28% higher odds of anaphylaxis.

Compared with previous studies, our work provides a detailed description of the allergological diagnostic pathway and subsequent management decisions in routine clinical practice. While previous studies primarily focused on desensitization outcomes [16,17], our study integrates diagnostic testing, risk stratification, and tailored re-exposure strategies, reflecting a comprehensive multidisciplinary approach. Indeed, enhanced premedication regimens are usually empirically administered rather than determined through randomized trials. Moreover, while premedication may attenuate the severity of hypersensitivity reactions, it does not reliably prevent anaphylaxis [7]. For this reason, accurate risk stratification of patients with suspected chemotherapy-related HRs is essential to minimize the risk of severe reactions while allowing continuation of optimal oncologic therapy [18]. These findings emphasize the clinical value of a multidisciplinary approach involving allergists and oncologists in the management of chemotherapy-related HRs. Collaborative decision-making allows accurate risk stratification and helps determine whether desensitization protocols or modified infusion strategies can be safely implemented, thereby reducing the need to discontinue first-line oncologic therapies. In our cohort, all patients undergoing re-exposure were managed following allergological assessment and individualized risk evaluation, and drug reintroduction using either desensitization protocols or enhanced premedication was successfully achieved in most cases. These findings support the concept that premedication alone should not be considered a protective strategy, but rather part of a broader, structured approach integrating clinical history, diagnostic testing, and multidisciplinary decision-making to ensure safe continuation of chemotherapy. Re-exposure to the culprit or related drug was feasible in nearly all patients, with a high tolerance rate (94%) using desensitization protocols or enhanced premedication. These results are consistent with growing evidence supporting desensitization as a safe and effective strategy to maintain essential oncologic therapies, even in patients with prior severe reactions [16,17]. The absence of severe adverse or hypersensitivity reactions supports the safety of tailored culprit or related drug reintroduction strategy. Furthermore, the identification of cumulative exposure as an independent predictor of anaphylaxis adds clinically relevant information to a field in which predictors of severe reactions remain not fully defined [19]. Our findings are consistent with previous studies describing platinum salts as the most frequently implicated agents in IgE-mediated chemotherapy-related HRs [20]. In addition, our findings support the reliability of skin testing in this setting. Reported rates of skin test positivity for platinum compounds in the literature range from 60% to 80% in selected cohorts, closely aligning with our observations [21]. Similarly, the lower diagnostic yield for taxanes and biologics parallels existing evidence suggesting lower rates of positivity for taxanes and biologics, likely reflecting non–IgE-mediated mechanisms, such as direct mast cell activation or complement activation-related pseudoallergy [22,23]. In addition, in our cohort, the median acute serum tryptase concentration appeared to be relatively low compared with values sometimes reported in severe anaphylaxis. However, serum tryptase elevation is not consistently observed in all hypersensitivity reactions and may be absent or modest in reactions related to chemotherapy agents, particularly when non-IgE-mediated mechanisms or mixed mechanisms are involved. Furthermore, baseline tryptase values were not available in our study, which limits the interpretation of relative increases, according to recommended diagnostic criteria [11].

From a clinical perspective, these findings highlight the importance of early referral to specialized allergy services after chemotherapy-related HRs, particularly in patients receiving multiple treatment cycles with high-risk agents. Multidisciplinary collaboration between allergists and oncologists is essential to ensure accurate diagnosis, appropriate risk stratification, and safe continuation of optimal oncologic therapy. The strong association between cumulative exposure and anaphylaxis highlights the need for heightened vigilance and risk assessment as treatment progresses. From a research standpoint, our findings reinforce the need for prospective studies integrating immunologic biomarkers, standardized diagnostic algorithms, and long-term oncologic outcomes for risk stratification and personalize management strategies. At the healthcare system level, the high success rate of drug reintroduction within a multidisciplinary care model highlights the critical role of structured collaboration between allergists and oncologists. Broader access to specialized drug allergy services could minimize unnecessary treatment modifications and help ensure equitable delivery of optimal oncologic care.

An important novel finding of this study is the trend toward an association between the number of prior chemotherapy cycles and the risk of anaphylaxis. Our multivariable analysis suggested that each additional cycle could increase the risk of anaphylaxis by approximately 28%, independent of age and history of previous drug allergy, although this observation should be interpreted cautiously. Although an increased risk of hypersensitivity reactions after repeated exposure has been previously described [24], particularly for platinum compounds, to which sensitization often develops after multiple infusions, cumulative exposure has not consistently been evaluated as an independent predictor of anaphylaxis across heterogeneous real-world populations undergoing allergological assessment. This supports the hypothesis that repeated exposure facilitates immune priming, particularly with platinum compounds, where sensitization often occurs after multiple infusions [24]. However, considering the relatively small sample size and the retrospective nature of the study, this finding should be interpreted cautiously. Moreover, the lack of association with previous drug allergies suggests that chemotherapy-induced anaphylaxis may be driven by a distinct immunopathological process rather than traditional atopic predisposition [25,26].

This study provides detailed real-world data on the diagnosis and management of chemotherapy-related HRs in a tertiary allergy centre, reflecting routine clinical practice rather than controlled experimental settings. A major strength is the systematic allergological evaluation applied to all included patients, using standardized skin testing and clearly defined clinical criteria, which allowed for consistent phenotyping of reactions and objective assessment of drug sensitization across different antineoplastic classes. In addition, structured multidisciplinary collaboration between allergists and oncologists enabled safe drug reintroduction in most cases, offering practical evidence for the feasibility and effectiveness of desensitization and enhanced premedication strategies in preserving essential oncologic treatments.

The study also specifically addressed cumulative drug exposure as a determinant of reaction severity. The identification of the number of prior chemotherapy cycles as an independent predictor of anaphylaxis contributes clinically relevant information to an area where risk stratification remains poorly defined, particularly outside highly selected cohorts [19]. Overall, our findings highlight the importance of integrating allergological expertise into oncologic care pathways to optimize treatment continuity and patient safety. From a practical perspective, our findings support the importance of integrating allergological evaluation into the clinical management of patients who develop suspected hypersensitivity reactions to chemotherapy. Early referral to specialized allergy services may facilitate accurate diagnosis, appropriate risk stratification, and the implementation of individualized strategies, thereby allowing continuation of optimal oncologic therapies whenever possible.

Several limitations should be acknowledged. The retrospective and monocentric design limits causal inference and may reduce generalizability to centres without dedicated allergy services. The relatively small sample size restricted the complexity of multivariable analyses and precluded the evaluation of additional potential predictors, such as immunologic biomarkers or drug-specific risk factors. In vitro testing was not systematically available for all patients, which may have influenced the diagnostic classification in selected cases. Finally, long-term oncologic outcomes after drug reintroduction were not assessed, limiting conclusions regarding the impact of allergological management on disease control or survival. Additionally, the relatively small number of anaphylaxis events limited the robustness of the multivariable logistic regression analysis. Indeed, the number of outcome events in our cohort may be insufficient to ensure stable estimates when multiple predictors are included in the model. Therefore, the results of the multivariable analysis should be interpreted cautiously and considered exploratory. These limitations may have influenced the interpretation of the observed associations, particularly regarding the relationship between cumulative chemotherapy exposure and the risk of anaphylaxis. Therefore, our findings should be interpreted with caution and considered exploratory, highlighting the need for prospective studies in larger and multicentre cohorts to confirm these observations. Finally, another limitation of this study is the marked predominance of female patients in the study population. This distribution largely reflects the underlying oncologic population referred to our centre, where a substantial proportion of patients were treated for gynecologic malignancies requiring platinum-based chemotherapy. However, this imbalance may limit the generalizability of the findings to male patients or to other oncologic populations with different treatment regimens.

## 5. Conclusions

We found that successful drug reintroduction occurred in all patients undergoing desensitization protocols and 92% undergoing enhanced premedication strategies. In addition, a trend toward an association between cumulative chemotherapy exposure and the occurrence of anaphylaxis was observed in our cohort. These findings highlight the importance of early allergological assessment, cumulative exposure as a potential risk marker, and the central role of desensitization protocols and premedication strategies in ensuring treatment continuity and patient safety. Future prospective, multicentre studies are needed to refine predictive models of reaction severity, integrate immunological biomarkers, and further evaluate long-term outcomes of desensitization and premedication strategies.

## Figures and Tables

**Table 1:** Baseline characteristics.

Characteristic	
Total, no.	42
Age, years—median [IQR]	57.5 [50.25–68]
Female—no. (%)	40 (95%)
Previous drug allergy history—no. (%) *	12 (29%)
Beta-lactams	4 (33%)
NSAIDS	2 (17%)
Gemcitabine	1 (8%)
Taxanes	3 (25%)
Doxorubicin	1 (8%)
Antiemetic drugs	3 (25%)
Allopurinol	1 (8%)
Oncologic diseases—no. (%)	
Gynaecological cancers	29 (69%)
Breast cancer	6 (14%)
Prostate cancer	2 (5%)
Lung cancer	3 (7%)
Colorectal cancer	2 (5%)
Clinical manifestations—no. (%)	
Erythematous rash	26 (62%)
Pruritus	10 (24%)
Urticaria	4 (10%)
Angioedema	8 (19%)
Dyspnoea	22 (52%)
Acute rhinitis	1 (2.4%)
Cardiovascular involvement	9 (21%)
Gastrointestinal symptoms	6 (14%)
Anaphylaxis	11 (26.2%)
Serum tryptase µg/L within two hours of reaction—median [IQR]	5.95 [4.4–9.5]

Abbreviations: no., number; IQR, interquartile range; NSAIDS, nonsteroidal anti-inflammatory drugs. * Percentages for specific drug allergy categories are calculated based on the subgroup of patients with a previous drug allergy history (n = 12). Included patients reported more than one drug allergy; therefore, the sum of individual allergy categories exceeds the number of patients.

**Table 2:** Summary of culprit drugs and chemotherapy reintroduction strategy.

Culprit Drug	Suspected Reactions n (%)	Skin Test Positive n (%)	Patients Requiring Drug Re-Exposure n (%)	Management Strategy	Successful Reintroduction n (%)
Platinum compounds	24 (57%)	17 (71%)			
Taxanes	10 (24%)	3 (30%)			
Biologic agents	5 (12%)	2 (40%)			
Total cohort	42	–	18 (43%)	Desensitization: 6 (33%) Enhanced premedication: 12 (67%)	17 (94%)

**Table 3:** Outcomes.

Outcomes	
Drug re-exposure—no. (%)	
Patients requiring re-exposure	18/42 (43%)
Desensitization protocol	6/18 (33%)
Enhanced premedication strategy	12/18 (67%)
Outcomes of drug re-exposure—no. (%)	
Successful reintroduction: desensitization	6/6 (100%)
Successful reintroduction: enhanced premedication	11/12 (92%)
Breakthrough adverse reactions during drug reintroduction	None
Skin test results—no. (%)	
Confirmed positivity among suspected reactions to platinum compounds	17/24 (71%)
Confirmed positivity among suspected reactions to taxanes	3/10 (30%)
Confirmed positivity among suspected reactions to biologic agents	2/5 (40%)
Anaphylaxis—no. (%)	
Patients with anaphylaxis	11/42 (26.2%)
Chemotherapy cycles before reaction: no anaphylaxis—median [IQR]	2 [1–4.8]
Chemotherapy cycles before reaction: anaphylaxis—median [IQR]	6 [2–9.5]

Abbreviations: no., number; IQR, interquartile range.

**Table 4:** Regression analysis of factors associated with anaphylaxis.

Variable	Unadjusted OR (95% CI)	*p*-Value	Adjusted OR (95% CI) *	*p*-Value
Number of chemotherapy cycles	1.29 (1.03–1.62)	0.028	1.28 (1.00–1.63)	0.05
Age	1.05 (0.98–1.12)	0.184	1.04 (0.96–1.12)	0.342
Previous drug allergy	1.12 (0.28–4.48)	0.873	0.85 (0.12–5.65)	0.867

Abbreviations: OR, odds ratio; CI, confidence interval. * Adjusted for age and previous history of drug allergy.

## Data Availability

Data are available upon request to the corresponding author.
